# 2,3,5-Triphenyl­pyrazine

**DOI:** 10.1107/S1600536808041627

**Published:** 2008-12-13

**Authors:** N. Anuradha, A. Thiruvalluvar, K. Pandiarajan, S. Chitra, R. J. Butcher

**Affiliations:** a PG Research Department of Physics, Rajah Serfoji Government College (Autonomous), Thanjavur-613 005, Tamil Nadu, India; b Department of Chemistry, Annamalai University, Annamalai Nagar 608 002, Tamil Nadu, India; c Department of Chemistry, Howard University, 525 College Street NW, Washington, DC 20059, USA

## Abstract

In the title mol­ecule, C_22_H_16_N_2_, the pyrazine ring deviates very slightly from planarity [maximum deviation 0.044 (3) Å], tending towards a twist-boat conformation. The phenyl ring at position 3 makes dihedral angles of 64.0 (2) and 45.8 (2)°, respectively, with the phenyl rings at positions 2 and 5. The dihedral angle between the phenyl rings at positions 2 and 5 is 49.7 (2)°. A C—H⋯π inter­action is found in the crystal structure, but no classical hydrogen bonds form.

## Related literature

For the biological properties of pyrazines, see: Foks *et al.* (2004 ▶); Premkumar & Govindarajan (2005 ▶); Sondhi *et al.* (2005 ▶).
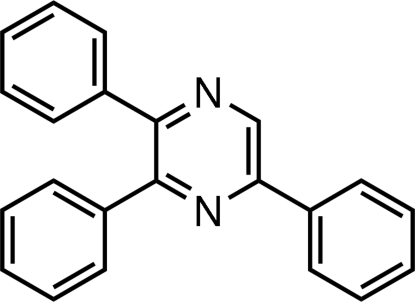


## Experimental

### 

#### Crystal data

C_22_H_16_N_2_
*M* *_r_* = 308.37
Orthorhombic, 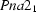

*a* = 15.563 (2) Å
*b* = 6.2005 (9) Å
*c* = 16.845 (3) Å
*V* = 1625.5 (4) Å^3^
*Z* = 4
Mo *K*α radiation
μ = 0.07 mm^−1^
*T* = 296 (2) K
0.44 × 0.35 × 0.21 mm

#### Data collection

Bruker APEXII CCD diffractometer
Absorption correction: multi-scan (*SADABS*; Bruker, 2004 ▶) *T*_min_ = 0.968, *T*_max_ = 0.985
17890 measured reflections
1730 independent reflections
1109 reflections with *I* > 2σ(*I*)
*R*_int_ = 0.091

#### Refinement

*R*[*F* ^2^ > 2σ(*F* ^2^)] = 0.050
*wR*(*F* ^2^) = 0.129
*S* = 1.02
1730 reflections
218 parameters
1 restraint
H-atom parameters constrained
Δρ_max_ = 0.13 e Å^−3^
Δρ_min_ = −0.13 e Å^−3^

### 

Data collection: *APEX2* (Bruker, 2004 ▶); cell refinement: *SAINT-NT* (Bruker, 2004 ▶); data reduction: *SAINT-NT*; program(s) used to solve structure: *SHELXS97* (Sheldrick, 2008 ▶); program(s) used to refine structure: *SHELXL97* (Sheldrick, 2008 ▶); molecular graphics: *ORTEP-3* (Farrugia, 1997 ▶); software used to prepare material for publication: *PLATON* (Spek, 2003 ▶).

## Supplementary Material

Crystal structure: contains datablocks global, I. DOI: 10.1107/S1600536808041627/wn2297sup1.cif

Structure factors: contains datablocks I. DOI: 10.1107/S1600536808041627/wn2297Isup2.hkl

Additional supplementary materials:  crystallographic information; 3D view; checkCIF report

## Figures and Tables

**Table 1:** Hydrogen-bond geometry (Å, °)

*D*—H⋯*A*	*D*—H	H⋯*A*	*D*⋯*A*	*D*—H⋯*A*
C53—H53⋯*Cg*^i^	0.93	2.98	3.909 (5)	174

Symmetry code: (i) 
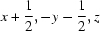
. *Cg* is the centroid of the C21–C26 phenyl ring.

**Fig. 1.**
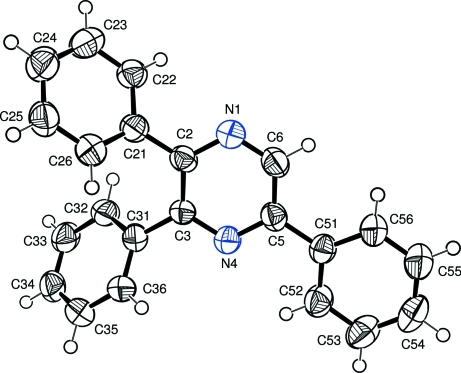
The molecular structure of the title compound, showing the atom-numbering scheme and displacement ellipsoids drawn at the 50% probability level. H atoms are shown as small spheres of arbitrary radius.

## supplementary crystallographic information

### Comment

Pyrazines are heterocycles which exhibit nutty, roasted or earth flavouring agents. 2-Cyanopyrazine derivatives show anticancer, anti-inflammatory and analgesic activities (Sondhi *et al.*, 2005). Pyrazine derivatives exhibit tuberculostatic activity (Foks *et al.*, 2004) and antimicrobial activity (Premkumar & Govindarajan, 2005).

In the title molecule, C_22_H_16_N_2_, (Fig. 1), the pyrazine ring deviates very slightly from planarity, tending towards a twist-boat conformation. The phenyl ring at position 3 makes dihedral angles of 64.0 (2)° and 45.8 (2)° with the phenyl rings at positions 2 and 5, respectively. The dihedral angle between the phenyl rings at positions 2 and 5 is 49.7 (2)°. A C53—H53···π interaction involving the phenyl (C21–C26) ring at position 2 is found in the crystal structure, but no classical hydrogen bonds.

### Experimental

To a homogeneous solution of benzil (1.05 g, 0.005 mol) and 1-phenylethanediamine dihydrochloride (1.04 g, 0.005 mol) in ethanol, sodium acetate trihydrate (2.04 g, 0.015 mol) was added. The precipitated sodium chloride was filtered off and the filtrate was refluxed for 2 h. On completion of the reaction, as indicated by TLC, the reaction mixture was poured into crushed ice and the resulting solid was filtered off and purified by column chromatography on silica gel. Elution with benzene: petroleum ether (4:1 v/v) at 333–353 K gave the product in pure form. Yield 1.6 g (80%).

### Refinement

H atoms were positioned geometrically and allowed to ride on their parent atoms, with C—H = 0.93 Å and *U*_iso_(H) = 1.2 times *U*_eq_(C). In the absence of significant anomalous scattering effects Friedel pairs have been merged.

### Crystal data

**Table:**

C_22_H_16_N_2_	*D*_x_ = 1.260 Mg m^−^^3^
*M**_r_* = 308.37	Melting point: 421 K
Orthorhombic, *P**n**a*2_1_	Mo *K*α radiation, λ = 0.71073 Å
Hall symbol: P 2c -2n	Cell parameters from 2955 reflections
*a* = 15.563 (2) Å	θ = 2.4–21.2°
*b* = 6.2005 (9) Å	µ = 0.07 mm^−^^1^
*c* = 16.845 (3) Å	*T* = 296 K
*V* = 1625.5 (4) Å^3^	Chunk, colourless
*Z* = 4	0.44 × 0.35 × 0.21 mm
*F*(000) = 648	

### Data collection

**Table:**

Bruker APEXII CCD diffractometer	1730 independent reflections
Radiation source: fine-focus sealed tube	1109 reflections with *I* > 2σ(*I*)
graphite	*R*_int_ = 0.091
φ and ω scans	θ_max_ = 26.5°, θ_min_ = 2.4°
Absorption correction: multi-scan (*SADABS*; Bruker, 2004)	*h* = −19→19
*T*_min_ = 0.968, *T*_max_ = 0.985	*k* = −7→7
17890 measured reflections	*l* = −20→19

### Refinement

**Table:**

Refinement on *F*^2^	Secondary atom site location: difference Fourier map
Least-squares matrix: full	Hydrogen site location: inferred from neighbouring sites
*R*[*F*^2^ > 2σ(*F*^2^)] = 0.050	H-atom parameters constrained
*wR*(*F*^2^) = 0.129	*w* = 1/[σ^2^(*F*_o_^2^) + (0.0653*P*)^2^ + 0.1081*P*] where *P* = (*F*_o_^2^ + 2*F*_c_^2^)/3
*S* = 1.02	(Δ/σ)_max_ = 0.001
1730 reflections	Δρ_max_ = 0.13 e Å^−^^3^
218 parameters	Δρ_min_ = −0.13 e Å^−^^3^
1 restraint	Extinction correction: *SHELXL97* (Sheldrick, 2008), Fc^*^=kFc[1+0.001xFc^2^λ^3^/sin(2θ)]^-1/4^
Primary atom site location: structure-invariant direct methods	Extinction coefficient: 0.015 (3)

### Special details

**Table:**

Geometry. Bond distances, angles *etc*. have been calculated using the rounded fractional coordinates. All su's are estimated from the variances of the (full) variance-covariance matrix. The cell e.s.d.'s are taken into account in the estimation of distances, angles and torsion angles
Refinement. Refinement of *F*^2^ against ALL reflections. The weighted *R*-factor *wR* and goodness of fit *S* are based on *F*^2^, conventional *R*-factors *R* are based on *F*, with *F* set to zero for negative *F*^2^. The threshold expression of *F*^2^ > 2σ(*F*^2^) is used only for calculating *R*-factors(gt) *etc*. and is not relevant to the choice of reflections for refinement. *R*-factors based on *F*^2^ are statistically about twice as large as those based on *F*, and *R*- factors based on ALL data will be even larger.

### Fractional atomic coordinates and isotropic or equivalent isotropic displacement parameters (Å^2^)

**Table:**

	*x*	*y*	*z*	*U*_iso_*/*U*_eq_	
N1	0.2355 (2)	0.1296 (5)	0.19242 (19)	0.0547 (12)	
N4	0.3436 (2)	−0.1252 (5)	0.10173 (19)	0.0487 (11)	
C2	0.2412 (3)	0.1612 (5)	0.1141 (2)	0.0463 (12)	
C3	0.3009 (3)	0.0395 (6)	0.0686 (2)	0.0447 (12)	
C5	0.3329 (3)	−0.1640 (6)	0.1791 (2)	0.0477 (12)	
C6	0.2822 (3)	−0.0252 (7)	0.2244 (3)	0.0557 (14)	
C21	0.1810 (3)	0.3188 (6)	0.0793 (2)	0.0480 (12)	
C22	0.1658 (3)	0.5110 (6)	0.1192 (3)	0.0547 (16)	
C23	0.1074 (3)	0.6577 (7)	0.0897 (3)	0.0670 (17)	
C24	0.0622 (3)	0.6125 (8)	0.0212 (3)	0.0717 (17)	
C25	0.0760 (3)	0.4218 (8)	−0.0185 (3)	0.0683 (19)	
C26	0.1359 (3)	0.2775 (8)	0.0110 (3)	0.0610 (17)	
C31	0.3243 (3)	0.0908 (7)	−0.0142 (2)	0.0500 (14)	
C32	0.3486 (3)	0.2977 (7)	−0.0358 (3)	0.0583 (16)	
C33	0.3772 (3)	0.3414 (7)	−0.1116 (3)	0.0653 (17)	
C34	0.3783 (3)	0.1810 (8)	−0.1676 (3)	0.0660 (17)	
C35	0.3525 (3)	−0.0229 (8)	−0.1474 (3)	0.0687 (17)	
C36	0.3261 (3)	−0.0692 (6)	−0.0714 (2)	0.0577 (16)	
C51	0.3770 (3)	−0.3528 (6)	0.2136 (2)	0.0483 (12)	
C52	0.4302 (3)	−0.4792 (7)	0.1662 (3)	0.0610 (17)	
C53	0.4682 (3)	−0.6620 (7)	0.1961 (3)	0.0730 (19)	
C54	0.4543 (3)	−0.7247 (8)	0.2734 (3)	0.0747 (19)	
C55	0.4017 (4)	−0.6012 (8)	0.3210 (3)	0.0770 (19)	
C56	0.3635 (3)	−0.4195 (7)	0.2911 (3)	0.0667 (17)	
H6	0.28114	−0.04259	0.27921	0.0669*	
H22	0.19510	0.54089	0.16601	0.0653*	
H23	0.09840	0.78757	0.11605	0.0803*	
H24	0.02234	0.71109	0.00186	0.0859*	
H25	0.04549	0.39011	−0.06447	0.0819*	
H26	0.14584	0.14928	−0.01612	0.0727*	
H32	0.34552	0.40842	0.00137	0.0699*	
H33	0.39584	0.47943	−0.12464	0.0783*	
H34	0.39640	0.21058	−0.21902	0.0788*	
H35	0.35281	−0.13135	−0.18555	0.0820*	
H36	0.30936	−0.20876	−0.05833	0.0693*	
H52	0.44015	−0.43949	0.11371	0.0731*	
H53	0.50365	−0.74446	0.16366	0.0872*	
H54	0.48007	−0.84883	0.29325	0.0894*	
H55	0.39220	−0.64112	0.37352	0.0927*	
H56	0.32749	−0.33888	0.32367	0.0800*	

### Atomic displacement parameters (Å^2^)

**Table:**

	*U*^11^	*U*^22^	*U*^33^	*U*^12^	*U*^13^	*U*^23^
N1	0.062 (2)	0.052 (2)	0.050 (2)	0.0055 (17)	0.0027 (16)	−0.0004 (16)
N4	0.0533 (19)	0.0458 (18)	0.047 (2)	0.0009 (15)	0.0024 (15)	0.0030 (15)
C2	0.057 (2)	0.042 (2)	0.040 (2)	−0.0052 (18)	0.0015 (17)	−0.0036 (18)
C3	0.055 (2)	0.039 (2)	0.040 (2)	0.0020 (18)	0.0004 (16)	−0.0025 (16)
C5	0.055 (2)	0.047 (2)	0.041 (2)	−0.0069 (18)	−0.0003 (17)	0.0022 (18)
C6	0.069 (3)	0.057 (2)	0.041 (2)	−0.002 (2)	−0.0020 (19)	−0.001 (2)
C21	0.051 (2)	0.047 (2)	0.046 (2)	−0.0008 (17)	0.0017 (18)	−0.0008 (18)
C22	0.060 (3)	0.050 (2)	0.054 (3)	0.000 (2)	0.0030 (19)	−0.0013 (19)
C23	0.067 (3)	0.055 (3)	0.079 (3)	0.011 (2)	0.007 (3)	0.001 (2)
C24	0.066 (3)	0.077 (3)	0.072 (3)	0.013 (3)	0.002 (3)	0.008 (3)
C25	0.067 (3)	0.085 (4)	0.053 (3)	0.012 (3)	−0.006 (2)	0.004 (2)
C26	0.067 (3)	0.062 (3)	0.054 (3)	0.002 (2)	−0.004 (2)	−0.002 (2)
C31	0.054 (2)	0.054 (3)	0.042 (2)	0.0078 (19)	−0.0010 (18)	0.0036 (18)
C32	0.077 (3)	0.047 (2)	0.051 (3)	0.003 (2)	0.009 (2)	0.002 (2)
C33	0.077 (3)	0.056 (3)	0.063 (3)	0.006 (2)	0.012 (2)	0.015 (2)
C34	0.076 (3)	0.076 (3)	0.046 (3)	0.015 (3)	0.009 (2)	0.014 (3)
C35	0.089 (3)	0.070 (3)	0.047 (3)	0.017 (3)	0.001 (2)	−0.006 (2)
C36	0.079 (3)	0.046 (2)	0.048 (3)	0.009 (2)	0.001 (2)	−0.004 (2)
C51	0.049 (2)	0.045 (2)	0.051 (2)	−0.0034 (18)	−0.0025 (18)	0.0050 (19)
C52	0.064 (3)	0.056 (3)	0.063 (3)	−0.001 (2)	0.005 (2)	0.013 (2)
C53	0.066 (3)	0.061 (3)	0.092 (4)	0.012 (2)	0.008 (3)	0.011 (3)
C54	0.075 (3)	0.056 (3)	0.093 (4)	0.007 (3)	−0.019 (3)	0.019 (3)
C55	0.098 (4)	0.072 (3)	0.061 (3)	0.010 (3)	−0.013 (3)	0.012 (3)
C56	0.085 (3)	0.064 (3)	0.051 (3)	0.013 (2)	−0.010 (2)	0.006 (2)

### Geometric parameters (Å, °)

**Table:**

N1—C2	1.337 (5)	C51—C56	1.386 (6)
N1—C6	1.319 (6)	C52—C53	1.374 (6)
N4—C3	1.340 (5)	C53—C54	1.376 (7)
N4—C5	1.336 (5)	C54—C55	1.378 (7)
C2—C3	1.421 (6)	C55—C56	1.370 (7)
C2—C21	1.475 (6)	C6—H6	0.9300
C3—C31	1.476 (5)	C22—H22	0.9300
C5—C6	1.395 (6)	C23—H23	0.9300
C5—C51	1.476 (6)	C24—H24	0.9300
C21—C22	1.389 (6)	C25—H25	0.9300
C21—C26	1.372 (6)	C26—H26	0.9300
C22—C23	1.379 (6)	C32—H32	0.9300
C23—C24	1.380 (7)	C33—H33	0.9300
C24—C25	1.375 (7)	C34—H34	0.9300
C25—C26	1.384 (7)	C35—H35	0.9300
C31—C32	1.386 (6)	C36—H36	0.9300
C31—C36	1.383 (5)	C52—H52	0.9300
C32—C33	1.379 (7)	C53—H53	0.9300
C33—C34	1.371 (7)	C54—H54	0.9300
C34—C35	1.370 (7)	C55—H55	0.9300
C35—C36	1.375 (6)	C56—H56	0.9300
C51—C52	1.392 (6)		
			
N1···N4	2.768 (4)	C34···H54^viii^	3.0900
N4···N1	2.768 (4)	C35···H54^viii^	2.9000
N1···H22	2.6600	C35···H56^v^	3.0600
N1···H35^i^	2.8800	C51···H22^ii^	3.0200
N4···H36	2.8000	C52···H23^vii^	3.0000
N4···H52	2.4700	C56···H6	2.6700
C5···C22^ii^	3.441 (6)	H6···C56	2.6700
C6···C34^iii^	3.587 (7)	H6···H56	2.1100
C6···C54^iv^	3.366 (7)	H22···N1	2.6600
C21···C32	3.253 (6)	H22···C5^iv^	2.8300
C22···C5^iv^	3.441 (6)	H22···C51^iv^	3.0200
C26···C32	3.405 (7)	H23···C52^ix^	3.0000
C26···C31	3.181 (7)	H25···C33^ix^	3.0900
C31···C26	3.181 (7)	H26···C3	2.8900
C32···C26	3.405 (7)	H26···C31	2.8000
C32···C21	3.253 (6)	H32···C2	2.9300
C34···C6^v^	3.587 (7)	H32···C21	2.9300
C35···C56^v^	3.576 (7)	H32···H52^iv^	2.5800
C54···C6^ii^	3.366 (7)	H35···N1^x^	2.8800
C56···C35^iii^	3.576 (7)	H36···N4	2.8000
C2···H32	2.9300	H52···N4	2.4700
C3···H26	2.8900	H52···H32^ii^	2.5800
C5···H22^ii^	2.8300	H54···C34^xi^	3.0900
C6···H56	2.6600	H54···C35^xi^	2.9000
C21···H32	2.9300	H55···C24^xii^	3.0000
C24···H55^vi^	3.0000	H56···C6	2.6600
C31···H26	2.8000	H56···H6	2.1100
C33···H25^vii^	3.0900	H56···C35^iii^	3.0600
			
C2—N1—C6	118.2 (4)	C54—C55—C56	120.1 (5)
C3—N4—C5	118.8 (3)	C51—C56—C55	121.8 (4)
N1—C2—C3	119.9 (3)	N1—C6—H6	119.00
N1—C2—C21	116.6 (3)	C5—C6—H6	119.00
C3—C2—C21	123.5 (3)	C21—C22—H22	120.00
N4—C3—C2	120.3 (3)	C23—C22—H22	120.00
N4—C3—C31	115.8 (4)	C22—C23—H23	120.00
C2—C3—C31	123.8 (3)	C24—C23—H23	120.00
N4—C5—C6	119.6 (4)	C23—C24—H24	120.00
N4—C5—C51	118.0 (3)	C25—C24—H24	120.00
C6—C5—C51	122.5 (3)	C24—C25—H25	120.00
N1—C6—C5	122.5 (4)	C26—C25—H25	120.00
C2—C21—C22	119.0 (3)	C21—C26—H26	119.00
C2—C21—C26	122.3 (4)	C25—C26—H26	119.00
C22—C21—C26	118.6 (4)	C31—C32—H32	119.00
C21—C22—C23	120.3 (4)	C33—C32—H32	120.00
C22—C23—C24	120.2 (4)	C32—C33—H33	120.00
C23—C24—C25	120.1 (4)	C34—C33—H33	120.00
C24—C25—C26	119.1 (5)	C33—C34—H34	120.00
C21—C26—C25	121.7 (4)	C35—C34—H34	120.00
C3—C31—C32	121.0 (4)	C34—C35—H35	120.00
C3—C31—C36	120.6 (4)	C36—C35—H35	120.00
C32—C31—C36	118.4 (4)	C31—C36—H36	120.00
C31—C32—C33	120.9 (4)	C35—C36—H36	120.00
C32—C33—C34	119.9 (4)	C51—C52—H52	120.00
C33—C34—C35	119.7 (5)	C53—C52—H52	120.00
C34—C35—C36	120.8 (4)	C52—C53—H53	120.00
C31—C36—C35	120.3 (4)	C54—C53—H53	120.00
C5—C51—C52	119.8 (3)	C53—C54—H54	120.00
C5—C51—C56	122.5 (4)	C55—C54—H54	120.00
C52—C51—C56	117.6 (4)	C54—C55—H55	120.00
C51—C52—C53	120.7 (4)	C56—C55—H55	120.00
C52—C53—C54	120.8 (4)	C51—C56—H56	119.00
C53—C54—C55	119.1 (5)	C55—C56—H56	119.00
			
C6—N1—C2—C3	−4.3 (6)	C2—C21—C22—C23	−177.5 (4)
C6—N1—C2—C21	173.4 (4)	C26—C21—C22—C23	−0.9 (7)
C2—N1—C6—C5	−3.9 (6)	C2—C21—C26—C25	176.3 (4)
C5—N4—C3—C2	−4.0 (6)	C22—C21—C26—C25	−0.2 (7)
C5—N4—C3—C31	172.3 (4)	C21—C22—C23—C24	1.4 (7)
C3—N4—C5—C6	−4.0 (6)	C22—C23—C24—C25	−0.8 (7)
C3—N4—C5—C51	176.9 (4)	C23—C24—C25—C26	−0.3 (7)
N1—C2—C3—N4	8.5 (6)	C24—C25—C26—C21	0.8 (7)
N1—C2—C3—C31	−167.5 (4)	C3—C31—C32—C33	174.6 (4)
C21—C2—C3—N4	−169.0 (4)	C36—C31—C32—C33	−2.6 (7)
C21—C2—C3—C31	15.0 (6)	C3—C31—C36—C35	−176.4 (4)
N1—C2—C21—C22	42.2 (6)	C32—C31—C36—C35	0.8 (7)
N1—C2—C21—C26	−134.2 (4)	C31—C32—C33—C34	2.8 (7)
C3—C2—C21—C22	−140.2 (4)	C32—C33—C34—C35	−1.3 (7)
C3—C2—C21—C26	43.4 (6)	C33—C34—C35—C36	−0.5 (7)
N4—C3—C31—C32	−126.1 (4)	C34—C35—C36—C31	0.7 (7)
N4—C3—C31—C36	51.0 (6)	C5—C51—C52—C53	−176.5 (4)
C2—C3—C31—C32	50.0 (7)	C56—C51—C52—C53	−0.6 (7)
C2—C3—C31—C36	−132.9 (5)	C5—C51—C56—C55	176.7 (5)
N4—C5—C6—N1	8.4 (7)	C52—C51—C56—C55	0.9 (7)
C51—C5—C6—N1	−172.6 (4)	C51—C52—C53—C54	0.3 (7)
N4—C5—C51—C52	2.3 (6)	C52—C53—C54—C55	−0.2 (7)
N4—C5—C51—C56	−173.5 (4)	C53—C54—C55—C56	0.5 (8)
C6—C5—C51—C52	−176.8 (4)	C54—C55—C56—C51	−0.9 (8)
C6—C5—C51—C56	7.5 (7)		

Symmetry codes: (i) −*x*+1/2, *y*+1/2, *z*+1/2; (ii) *x*, *y*−1, *z*; (iii) −*x*+1/2, *y*−1/2, *z*+1/2; (iv) *x*, *y*+1, *z*; (v) −*x*+1/2, *y*+1/2, *z*−1/2; (vi) −*x*+1/2, *y*+3/2, *z*−1/2; (vii) *x*+1/2, −*y*+1/2, *z*; (viii) −*x*+1, −*y*−1, *z*−1/2; (ix) *x*−1/2, −*y*+1/2, *z*; (x) −*x*+1/2, *y*−1/2, *z*−1/2; (xi) −*x*+1, −*y*−1, *z*+1/2; (xii) −*x*+1/2, *y*−3/2, *z*+1/2.

### Hydrogen-bond geometry (Å, °)

**Table:**

*D*—H···*A*	*D*—H	H···*A*	*D*···*A*	*D*—H···*A*
C53—H53···Cg^xiii^	0.93	2.98	3.909 (5)	174

Symmetry codes: (xiii) *x*+1/2, −*y*−1/2, *z*.

## References

[bb1] Bruker (2004). *APEX2*, *SAINT-NT* and *SADABS* Bruker AXS Inc., Madison, Wisconsin, USA.

[bb2] Farrugia, L. J. (1997). *J. Appl. Cryst.* **30**, 565.

[bb3] Foks, H., Trapkaoska, I., Janowiec, M., Zwolska, Z. & Augustynowicz-Kopec, E. (2004). *Chem. Heterocycl. Cmpd*, **40**, 1185–1193.

[bb4] Premkumar, T. & Govindarajan, S. (2005). *World J. Microbiol. Biotechnol.* **21**, 479–480.

[bb5] Sheldrick, G. M. (2008). *Acta Cryst.* A**64**, 112–122. 10.1107/S0108767307043930 18156677

[bb6] Sondhi, S. M., Singh, N., Rajvanshi, S. & Johar, M. (2005). *Indian J. Chem.* **44B**, 387–399.

[bb7] Spek, A. L. (2003). *J. Appl. Cryst.* **36**, 7–13.

